# Crohn’s disease in coastal China: epidemiological profile and determinants of diagnostic delay in the Yancheng cohort

**DOI:** 10.3389/fphys.2025.1643597

**Published:** 2025-08-25

**Authors:** Shengnan Jin, Maozhen Zhang, Zixuan Zhou, Yanping Hao, Lin Wang, Su Xu

**Affiliations:** ^1^ Department of Anorectal Surgery, Yancheng TCM Hospital Affiliated to Nanjing University of Chinese Medicine, Yancheng, China; ^2^ Department of Gastroenterology, Yancheng NO.1 People’s Hospital, Yancheng, China

**Keywords:** Crohn’s disease, epidemiology, diagnostic delay, China, Yancheng

## Abstract

**Background and aims:**

Crohn’s disease (CD) exhibits escalating incidence in China, particularly in coastal regions undergoing rapid industrialization. We aim to investigate the epidemiological characteristics of CD and identify factors influencing diagnostic delay in the Yancheng region of China.

**Methods:**

A cross-sectional analysis was conducted on CD patients from two tertiary medical centers (Yancheng TCM Hospital Affiliated to Nanjing University of Chinese Medicine and Yancheng No.1 People’s Hospital) between October 2021 and October 2024. We calculated crude prevalence and incidence rates, and performed logistic regression analyses to identify predictors of diagnostic delay.

**Results:**

Among 200 enrolled CD patients (male-to-female ratio: 2.07:1), the crude prevalence and incidence rates were 17.48/100,000 and 2.3/100,000, respectively. Diagnostic delay occurred in 48% (96/200) of cases, with a median delay duration of 23 months (IQR: 12–48; maximum: 300 months). Multivariate analysis identified advanced age at diagnosis as an independent risk factor for delay (OR = 1.035, 95% CI: 1.010–1.060, *P* = 0.005), while abdominal pain served as a protective factor (OR = 2.088, 95% CI: 1.136–3.838, *P* = 0.018). Diagnostic delay duration correlated positively with age at diagnosis (r = 0.302, *P* = 0.003) and frequency of refrigerated food consumption (r = 0.219, *P* = 0.032). No significant associations were observed between delay duration and complications such as intestinal obstruction, perforation, or surgical intervention *(P* > 0.05).

**Conclusion:**

The Yancheng region exhibits higher CD prevalence and incidence rates compared to national averages, with pronounced diagnostic delays. These findings highlight the need for targeted interventions to improve early diagnosis and mitigate healthcare burdens.

## 1 Introduction

Crohn’s disease (CD), a chronic idiopathic inflammatory bowel disorder affecting the entire gastrointestinal tract (predominantly the terminal ileum and proximal colon), exhibits marked global epidemiological heterogeneity. CD pathogenesis arises from multifactorial interactions involving impaired intestinal barrier function, dysregulation of both innate and adaptive immune responses, and alterations in gut microbial ecology. These interconnected pathological mechanisms collectively drive the characteristic chronic transmural inflammation observed in this condition (Roda et al., 2020). While Australia reports the highest incidence (29.3 per 10^5^ person-years) and Europe/Canada demonstrate prevalence peaks (322.0 and 319.0 per 10^5^, respectively) (Ng et al., 2017), Asia is experiencing an accelerating epidemiological transition. Rapidly rising CD rates in this region are hypothesized to reflect lifestyle shifts, dietary modifications, and environmental exposures (GBD, 2017 Inflammatory Bowel Disea se Collaborators, 2020). In China, national incidence surged from 0.46/10^5^ (2010-2013) (Cui and Yuan, 2018) to 0.71/10^5^ in urban areas by 2016 (Xu et al., 2023), underscoring evolving disease dynamics. Notably, China’s vast geographical diversity—encompassing distinct climate zones, socioeconomic gradients, and genetic/environmental interactions—drives heterogeneous epidemiological patterns (Cui and Yuan, 2018). Current CD research predominantly focuses on metropolitan hubs (Shanghai, Guangzhou, Wuhan) and high-prevalence provinces (Guangdong, Zhejiang, Taiwan) (Xu et al., 2023; Wang et al., 2013; Ng et al., 2016; Chen et al., 2025), leaving coastal-industrial regions like Yancheng critically underrepresented. Situated in Jiangsu Province within the Yangtze River Delta, Yancheng exemplifies unique coastal-industrial characteristics: high humidity, seafood-rich diets, rapid industrialization, and documented water quality concerns. These factors may synergistically modulate CD risk, yet systematic epidemiological data remain absent. This study pursues dual objectives: 1) to delineate CD epidemiological profiles in Yancheng, addressing critical data gaps for coastal China; 2) to identify determinants of diagnostic delay, a pervasive issue compromising therapeutic efficacy. Prolonged diagnostic intervals correlate with reduced quality of life, delayed biologic initiation, and irreversible bowel damage. Early use of biologics reduces CD-related surgery rates and the risk of hormone dependence (Lujan et al., 2024). Concurrently, early post-diagnosis initiation of biologic agents has been evidenced to reduce cumulative healthcare expenditures while mitigating long-term healthcare resource utilization (Ungaro et al., 2024). Consequently, this study incorporates a focused investigation into diagnostic delay, analyzing region-specific risk factors. We aim to inform targeted prevention strategies and optimize clinical management.

## 2 Methods

This study protocol received ethical clearance from the Ethics Committee of Yancheng TCM Hospital Affiliated to Nanjing University of Chinese Medicine (Approval No. KY241101) in November 2024. Following rigorous evaluation by two board-certified inflammatory bowel disease (IBD) specialists and acquisition of informed consent, 200 confirmed CD patients were retrospectively enrolled in this cohort study. The sample size required for this epidemiological study was calculated using the standard formula for prevalence surveys:

$$n=\frac{Z_{1-\alpha /2}^{2}\times p\left(1-p\right)}{d^{2}}\times Deff$$

Where: *Z*_1−*α*/2_ = Z-value for the desired confidence level, *p* = Expected prevalence of Crohn’s disease, *d* = Margin of error, Deff = Design effect accounting for cluster sampling.

Utilizing demographic data derived from the Yancheng Population Census, we calculated disease frequency metrics as follows:

Prevalence was computed as the proportion of individuals with existing cases relative to the permanent resident population at a specified reference date:

$$\text{Prevalence}=\frac{Numberofprevalentcases}{Size\;of\;permanent\;resident\;population}\times 100,000$$

Incidence was defined as the number of newly confirmed cases occurring during the observation period divided by the at-risk population:

$$Incidence=\frac{Numberofincidentcases}{Size\;of\;exposure\;population\;at\;risk}\times 100,000$$

Diagnostic delay was defined as time period (in months) from first symptoms to CD diagnosis (Schoepfer et al., 2013).

### 2.1 Patient recruitment

Given the centralized healthcare-seeking pattern of CD patients in Yancheng, where a large proportion of regional cases are managed at two tertiary centers (Yancheng Affiliated Hospital of Nanjing University of Chinese Medicine and Yancheng NO.1 People’s Hospital), we consecutively enrolled all confirmed CD patients attending these institutions from October 2021 to October 2024. Diagnosis was validated through Chinese clinical practice guideline on the management of Crohn’s disease or ECCO Guidelines on the Prevention, Diagnosis, and Management of Infections in Inflammatory Bowel Disease (Inflammatory Bowel Disease Group et al., 2024; Kucharzik et al., 2021). Exclusion criteria comprised incomplete clinical documentation, cognitive impairment affecting consent capacity, concurrent participation in interventional trials or Withdrawal of informed consent. Control subjects were systematically recruited from the same clinical settings, with comprehensive exclusion criteria encompassing inflammatory bowel disease (Crohn’s disease or ulcerative colitis), autoimmune disorders (e.g., rheumatoid arthritis, systemic lupus erythematosus), gastrointestinal malignancies, and other forms of colitis (including infectious, ischemic, or microscopic colitis).

### 2.2 Data collection

Referring to the relevant literature (Xu et al., 2023), we formulated the “Yancheng Region Crohn’s Disease Questionnaire” to register the clinical data of patients in Yancheng region in detail, including demographic information (age, gender, occupation, residential area, education level, etc.), disease characteristics (Symptom duration, Montreal classification, therapeutic interventions, complication profiles, etc.), as well as potentially influencing factors (family history, smoking, diet, history of antibiotic use, gastrointestinal infections, pharmacological history, parasitic infections, measles, rubella and mumps, etc.).

### 2.3 Statistical analysis

All analyses were conducted using SPSS Statistics 27.0 (IBM Corp.). Continuous variables underwent normality assessment via Shapiro-Wilk tests, supplemented by visual inspection of histograms and Q-Q plots. Normally distributed data were expressed as mean ± standard deviation (SD) and analyzed using independent t-tests (two-group comparisons) or one-way ANOVA (multi-group comparisons). Non-parametric variables were reported as median (interquartile range, IQR) with Mann-Whitney U tests for between-group analyses. Categorical variables were presented as frequencies (%) and compared via χ^2^ or Fisher’s exact tests. Multivariate logistic regression analyses were implemented to identify independent determinants of Crohn’s disease development and diagnostic delay. Variables demonstrating preliminary associations (*P* ≤ 0.05) in univariate screening were incorporated into the logistic regression models, with computation of odds ratios (ORs) and corresponding 95% confidence intervals (95% CIs). Spearman’s rank correlation coefficients (r) quantified temporal relationships between diagnostic delay duration and clinical covariates, with Bonferroni correction applied for multiple comparisons. Statistical significance thresholds were defined as *P* < 0.05 (two-tailed) and *P* < 0.01 (high significance).

## 3 Results

### 3.1 Incidence and prevalence of CD in Yancheng

Based on municipal demographic data (resident population: 7.1 million), Yancheng exhibited a crude CD prevalence of 17.48 cases per 100,000 population, with an annual incidence rate of 2.30/100,000 in 2024.

### 3.2 Demographic profile of the CD cohort

**FIGURE 1:**
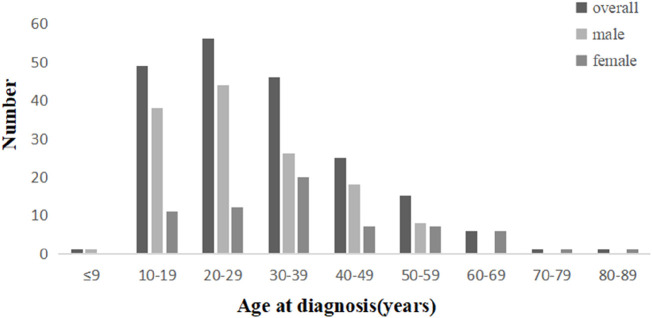
Distribution of age at diagnosis of CD patients in Yancheng region. Bar graph showing the number of medical diagnoses across different age groups divided by gender: overall, male, and female. Ages range from under 9 to 80–89 years. The highest number of diagnoses occurs in the 10–19 and 20–29 age ranges. Sex-specific disparities were observed (male peak: 20–29 years vs. female peak: 30–39 years.

**FIGURE 2:**
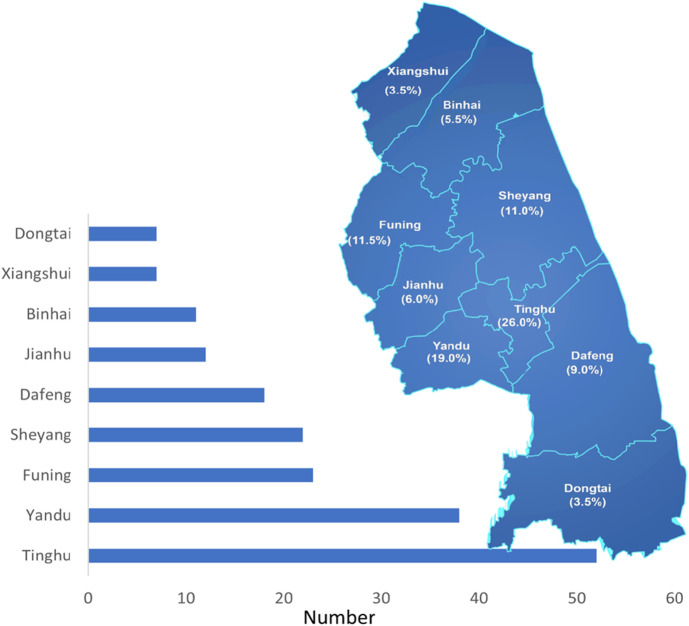
Distribution of CD patients in Yancheng region. Map of a region divided into labeled areas, each with percentages. Tinghu has the highest percentage at 26.0%, followed by Yandu at 19.0%, and Xiangshui has the lowest percentage at 3.5%.

This study enrolled 200 patients with CD, excluding 14% of screened cases. The cohort comprised predominantly male patients (male-to-female ratio: 2.07:1) with a median diagnosis age of 29 years (IQR 20–39; range 12–80, exhibiting a unimodal age distribution peaking at 20–29 years overall, though sex-specific disparities were observed (male peak: 20–29 years vs. female peak: 30–39 years; Figure 1). Geospatial analysis revealed concentrated case distribution in Tinghu District (26.0%, n = 52) and minimal representation in Xiangshui County (3.5%, n = 7; Figure 2). The cohort predominantly comprised Han Chinese (99.5%), married individuals (55.5%, n = 111), and residents of small cities (towns) (67.5%, n = 135). Socioeconomically, 47.0% (n = 94) had secondary education or below, 57.0% (n = 114) reported monthly incomes below 3 200 CNY, and 58.5% (n = 117) were covered by urban employee basic medical insurance. Occupational profiling identified non-working status (retirees/homemakers/unemployed) in 49.0% (n = 98), with white-collar workers comprising 40.0% (n = 80) and moderate occupational intensity reported by 63.0% (n = 126) (Table 1).

**TABLE 1:** Comparison of demographic characteristics between the two patient groups.

Variables	Delayed-diagnosis group (n = 96)	Non-delayed group (n = 104)	χ^2^/U	*P* Value
Age at diagnosis (years)	33.5 (22.0,44.5)	26.5 (18.5,33.0)	3504.5	<0.01
Gender			1.319	0.251^a^
Male	61 (63.5)	74 (71.2)		
Female	35 (36.5)	30 (28.8)		
Marital status			4.908	0.256^d^
Married	61 (63.5)	50 (48.1)		
Never married	32 (33.3)	49 (47.1)		
Divorced	2 (2.1)	3 (2.9)		
Widowed	1 (1.0)	2 (1.9)		
Ethnicity			-	0.48^b^
Han	95 (99.9)	104 (100.0)		
Others	1 (1.0)	0		
District			12.855	0.169^d^
Tinghu	24 (25.0)	28 (26.9)		
Yandu	21 (21.9)	17 (16.3)		
Dafeng	6 (6.3)	12 (11.5)		
Dongtai	2 (2.1)	5 (4.8)		
Jianhu	9 (9.4)	3 (2.9)		
Sheyang	10 (10.4)	12 (11.5)		
Binhai	2 (2.1)	9 (8.7)		
Funing	13 (13.5)	10 (9.6)		
Xiangshui	3 (3.1)	4 (3.8)		
Others	6 (6.3)	4 (3.8)		
Residential area in the past 5 years			6.896	0.032^a^
Small cities (towns)	64 (66.7)	71 (68.3)		
Large and medium-sized cities	13 (13.5)	24 (23.1)		
Rural area	19 (19.8)	9 (8.7)		
Occupation			11.6	0.041^d^
Worker	16 (16.7)	18 (17.3)		
Farmers or herdsmen	9 (9.4)	2 (1.9)		
Office-worker	17 (17.7)	24 (23.1)		
Civil servant	1 (1.0)	2 (1.9)		
Self-employed occupational status	10 (10.4)	3 (2.9)		
Non-working status	43 (44.8)	55 (52.9)		
Occupational characteristics			0.781	0.677^a^
Manual labor	31 (32.3)	28 (26.9)		
Combined physical-cognitive work	29 (30.2)	32 (30.8)		
Non-manual labor	36 (37.5)	44 (42.3)		
Occupational tension			1.138	0.566^a^
Low	22 (22.9)	21 (20.2)		
Moderate	57 (59.4)	69 (66.3)		
High	17 (17.7)	14 (13.5)		
Monthly income			0.242	0.623^a^
<3200 CNY	53 (55.2)	61 (58.7)		
≥3200 CNY	43 (44.8)	43 (41.3)		
Types of medical insurance			1.989	0.371^d^
Urban Employee Basic Medical Insurance	61 (63.5)	56 (53.8)		
Urban and Rural Resident Basic Medical Insurance	34 (35.4)	47 (45.2)		
Self-payment	1 (1.0)	1 (1.0)		
Education level			3.939	0.14^d^
secondary education or below	52 (54.2)	42 (40.4)		
college diploma/bachelor’s degree	42 (43.8)	60 (57.7)		
postgraduate education	2 (2.1)	2 (1.9)		

^a^ Pearson’s chi-square test.

^b^ Continuity correction.

^c^ Likelihood ratio chi-square.

^d^ Fisher’s exact test.

### 3.3 Clinical characteristics and disease phenotypes

The cohort comprised 200 CD patients with a median disease duration of 6 years (IQR 4–10), predominantly presenting with intestinal manifestations (n = 176, 88.0%), including abdominal pain (n = 122), diarrhea (n = 124), hematochezia (n = 44), intestinal obstruction (n = 51), and perforation (n = 22). Extraintestinal manifestations affected 40.0% (n = 80) of patients, involving dermatological (n = 36), arthralgia (n = 33), ocular (n = 17), and hepatobiliary systems (n = 10). Disease localization followed Montreal classification: ileal (L1: 39.5%, n = 79), colonic (L2: 12.5%, n = 25), ileocolonic (L3: 47.0%, n = 94), and upper gastrointestinal involvement (L4: 11.5%, n = 23). Disease behavior was non-stricturing, non-penetrating (B1: 55.0%, n = 110), stricturing (B2: 31.0%, n = 62), or penetrating (B3: 14.0%, n = 28), with perianal complications documented in 60.5% (n = 121) of cases (Table 2).

**TABLE 2:** Comparison of clinical characteristics between the two patient groups.

Variables	Delayed-diagnosis group (n = 96)	Non-delayed group (n = 104)	χ^2^/U	*P* Value
Disease duration (years)	7 (4,12)	6 (4,9)	4335.5	0.107
Age at diagnosis (years)			9.472	0.09^a^
A1 (≤16)	11 (11.5)	16 (15.4)		
A2 (17-40)	56 (58.3)	75 (72.1)		
A3 (≥41)	29 (30.2)	13 (12.5)		
Disease location			4.500	0.212^d^
L1 (ileal)	43 (44.8)	36 (34.6)		
L2 (colonic)	11 (11.5)	14 (13.5)		
L3 (ileocolonic)	42 (43.8)	52 (50.0)		
L4 (upper gastrointestinal tract)	0	2 (1.9)		
Disease behavior			2.597	0.273^a^
B1 (non-stricturing,non-penetrating)	49 (51.0)	61 (58.7)		
B2 (stricturing)	35 (36.5)	27 (26.0)		
B3 (penetrating)	12 (12.5)	16 (15.4)		
Perianal complications	53 (55.2)	68 (65.4)	2.163	0.141^a^
Intestinal manifestations				
Abdominal pain	68 (70.8)	54 (51.9)	7.504	0.006^a^
Diarrhea	59 (61.5)	65 (62.5)	0.023	0.879^a^
Hematochezia	18 (18.8)	26 (25.0)	1.136	0.286^a^
Intestinal obstruction	27 (28.1)	24 (23.1)	0.670	0.413^a^
Intestinal perforation	11 (11.5)	11 (10.6)	0.040	0.842^a^
Others	2 (2.1)	3 (2.9)	0.00	1.00^c^
Extraintestinal manifestations				
Dermatological system	18 (18.8)	18 (17.3)	0.070	0.791^a^
Arthralgia system	13 (13.5)	20 (19.2)	1.173	0.279^a^
Ocular system	8 (8.3)	9 (8.7)	0.007	0.935^a^
Hepatobiliary system	3 (3.1)	7 (6.7)	0.713	0.399^c^
Others	4 (4.2)	5 (4.8)	0.00	1.00^c^

^a^ Pearson’s chi-square test.

^b^ Continuity correction.

^c^ Likelihood ratio chi-square.

^d^ Fisher’s exact test.

### 3.4 Treatment patterns and therapeutic interventions

Among the 200 CD patients analyzed, biologic agents constituted the predominant therapeutic strategy (n = 178, 89.0%), followed by surgical intervention (n = 50, 25.0%) and conventional therapies including 5-Aminosalicylic acid (5-ASA), corticosteroids, or immunosuppressants (n = 40, 20.0%), with multiple treatment modalities employed in progressive cases (Table 3).

**TABLE 3:** Comparison of therapeutic interventions between the two patient groups.

Therapeutic interventions	Delayed-diagnosis group (n = 96)	Non-delayed group (n = 104)	χ^2^/U	*P* Value
None	0	1 (1.0)	-	1.000^b^
Conventional medicine	22 (22.9)	18 (17.3)	0.982	0.322^a^
Biologic agents	81 (84.4)	97 (93.3)	4.034	0.045^a^
Surgical intervention	29 (30.2)	21 (20.2)	2.671	0.102^a^

^a^ Pearson’s chi-square test.

^b^ Continuity correction.

^c^ Likelihood ratio chi-square.

^d^ Fisher’s exact test.

### 3.5 Multivariable determinants of Crohn’s disease risk in coastal China

**FIGURE 3:**
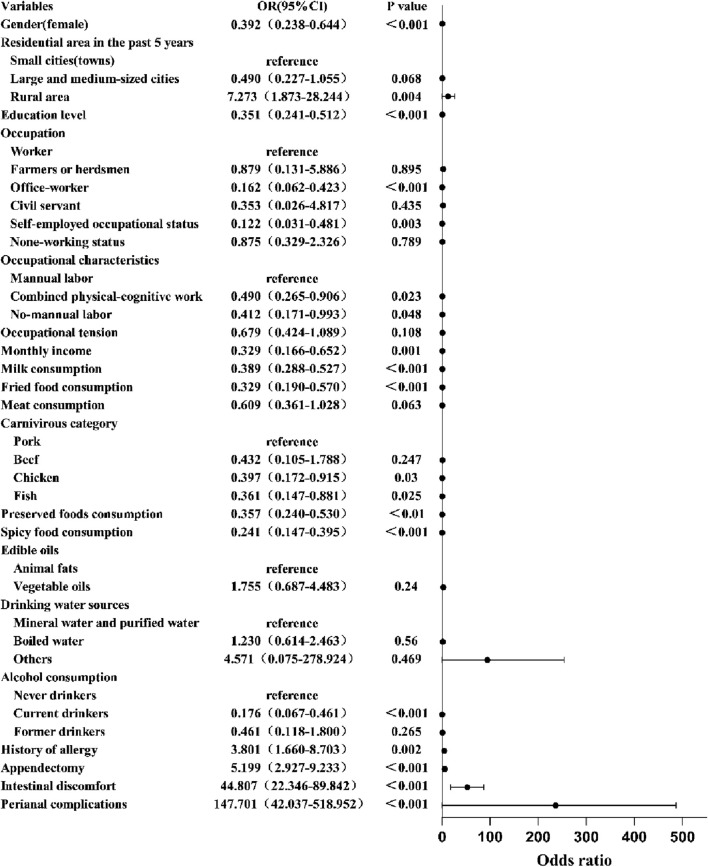
Multivariable analysis of factors influencing Crohn’s disease pathogenesis. The forest plot displays odds ratios (OR) and confidence intervals (CI) for various variables affecting an unspecified outcome. Significant associations, marked by dots and horizontal lines, show factors like gender, education level, and conditions like intestinal discomfort and perianal complications are highly significant (*p* < 0.001).

In this case-control study comprising 200 CD cases and 200 matched controls, univariate analysis identified 20 significant determinants of CD development, spanning sociodemographic factors (gender, recent residential area [past 5 years], education level, occupation, occupational characteristics, occupational stress levels, monthly income), dietary patterns (meat consumption frequency/types, edible oil classes, milk intake, fried/preserved/spicy food consumption, drinking water sources), lifestyle exposures (alcohol consumption), clinical history (history of allergy, appendectomy), and disease-specific features (intestinal symptoms, perianal complications), as detailed in Table 4. Multivariable logistic regression adjusted for age and genetic confounders identified intestinal symptoms (OR = 44.807, 95%CI: 22.346-89.842), perianal complications (OR = 147.701, 95%CI:42.037-518.952), appendectomy (OR = 5.199, 95%CI: 2.927-9.233), history of allergy (OR = 3.801, 95%CI: 1.660-8.703),and rural residence history (OR = 7.273, 95%CI: 1.873-28.244) as independent risk determinants, while protective associations emerged across sociodemographic (female gender, higher education, office-worker, self-employed occupational status, non-working status, occupations devoid of physical exertion, elevated income) and dietary domains (milk consumption, chicken/fish intake, preserved foods, moderate fried/spicy food intake, alcohol consumption) (Figure 3).

**TABLE 4:** Univariate analysis of factors influencing Crohn’s disease pathogenesis.

Variables	CD Group (n = 200)	Healthy controls (n = 200)	χ^2^/U	*P* Value
Age at diagnosis(years)	29 (20,39)	29 (24,39)	17,790.5	0.056
Gender			23.322	<0.01^a^
Male	135 (67.5)	87 (43.5)		
Female	65 (32.5)	113 (56.5)		
Marital status			4.933	0.177^c^
Married	111 (55.5)	91 (45.5)		
Never married	81 (40.5)	103 (51.5)		
Divorced	5 (2.5)	4 (2.0)		
Widowed	3 (1.5)	2 (1.0)		
Ethnicity			-	1.00^d^
Han	199 (99.5)	199 (99.5)		
Others	1 (0.5)	1 (0.5)		
Residential area in the past 5 years			23.457	<0.01^a^
Small cities (towns)	135 (67.5)	136 (68.0)		
Large and medium-sized cities	37 (18.5)	60 (30.0)		
Rural area	28 (14.0)	4 (2.0)		
Education level			72.403	<0.01^a^
secondary education or below	94 (47.0)	46 (23.0)		
college diploma/bachelor’s degree	102 (51.0)	88 (44.0)		
postgraduate education	4 (2.0)	66 (33.0)		
Types of medical insurance			5.405	0.067^c^
Urban Employee Basic Medical Insurance	117 (58.5)	97 (48.5)		
Urban and Rural Resident Basic Medical Insurance	81 (40.5)	97 (48.5)		
Self-payment	2 (1.0)	6 (3.0)		
Occupation			35.131	<0.001^c^
Worker	34 (17.0)	15 (7.5)		
Farmers or herdsmen	11 (5.5)	2 (1.0)		
Office-worker	41 (20.5)	84 (42.0)		
Civil servant	3 (1.5)	2 (1.0)		
Self-employed occupational status	13 (6.5)	22 (11.0)		
Non-working status	98 (49.0)	75 (37.5)		
Occupational characteristics			10.401	0.006^a^
Manual labor	59 (29.5)	32 (16.0)		
Combined physical-cognitive work	61 (30.5)	71 (35.5)		
Non-manual labor	80 (40.0)	97 (48.5)		
Occupational tension			29.387	<0.001^a^
Low	43 (21.5)	42 (21.0)		
Moderate	126 (63.0)	81 (40.5)		
High	31 (15.5)	77 (38.5)		
Monthly income			21.333	<0.001^a^
<3200 CNY	114 (57.0)	68 (34.0)		
≥3200 CNY	86 (43.0)	132 (66.0)		
Regularity of meal intake			5.895	0.052^a^
Regular	127 (63.5)	115 (57.5)		
1-2 irregular meals/week	49 (24.5)	43 (21.5)		
≥3 irregular meals/week	24 (12.0)	42 (21.0)		
Meat consumption			19.928	<0.001^a^
Never	11 (5.5)	0		
1-2 times/week	57 (28.5)	35 (17.5)		
≥3 times/week	132 (66.0)	165 (82.5)		
Carnivirous category			38.308	<0.001^c^
Pork	150 (75.0)	98 (49.0)		
Beef	8 (4.0)	19 (9.5)		
Chicken	21 (10.5)	47 (23.5)		
Fish	17 (8.5)	36 (18.0)		
Others	4 (2.0)	0		
Edible oils			5.530	0.019^a^
Animal fats	8 (4.0)	20 (10.0)		
Vegetable oils	192 (96.0)	180 (90.0)		
Eggs consumption			5.280	0.071^a^
Never	9 (4.5)	4 (2.0)		
1-2 times/week	61 (30.5)	80 (40.0)		
≥3 times/week	130 (65.0)	116 (58.0)		
Milk consumption			75.380	<0.001^a^
Never	141 (70.5)	55 (27.5)		
1-2 times/week	34 (17.0)	69 (34.5)		
≥3 times/week	25 (12.5)	76 (38.0)		
Fried food consumption			33.079	<0.001^a^
Never	112 (56.0)	56 (28.0)		
1-2 times/week	78 (39.0)	121 (60.5)		
≥3 times/week	10 (5.0)	23 (11.5)		
Preserved foods consumption			35.438	<0.001^a^
Never	128 (64.0)	72 (36.0)		
1-2 times/week	68 (34.0)	108 (54.0)		
≥3 times/week	4 (2.0)	20 (10.0)		
Spicy food consumption			114.767	<0.001^a^
Never	153 (76.5)	47 (23.5)		
1-2 times/week	36 (18.0)	95 (47.5)		
≥3 times/week	11 (5.5)	58 (29.0)		
Refrigerated food consumption			5.255	0.072^a^
Never	58 (29.0)	39 (19.5)		
1-2 times/week	88 (44.0)	105 (52.5)		
≥3 times/week	54 (27.0)	56 (28.0)		
Heavy sugar consumption			1.913	0.384^a^
Never	53 (26.5)	53 (26.5)		
1-2 times/week	118 (59.0)	108 (54.0)		
≥3 times/week	29 (14.5)	39 (19.5)		
Marine and freshwater aquatic products			5.192	0.075^a^
Never	124 (62.0)	102 (51.0)		
1-2 times/week	70 (35.0)	88 (44.0)		
≥3 times/week	6 (3.0)	10 (5.0)		
Vegetables and fruits consumption			1.581	0.454^a^
Never	8 (4.0)	5 (2.5)		
1-2 times/week	68 (34.0)	78 (39.0)		
≥3 times/week	124 (62.0)	117 (58.5)		
Drinking water sources			23.828	<0.001^a^
Boiled water	143 (71.5)	102 (51.0)		
Mineral water and purified water	49 (24.5)	95 (47.5)		
Others	8 (4.0)	3 (1.5)		
Dietary patterns			2.452	0.294^a^
Vegetarian diet	20 (10.0)	17 (8.5)		
Meat-based diet	11 (5.5)	19 (9.5)		
Omnivorous diet	169 (84.5)	164 (82.0)		
Smoking			7.140	0.068^c^
Never smokers	179 (89.5)	170 (85.0)		
Current smokers	10 (5.0)	23 (11.5)		
Former smokers	7 (3.5)	3 (1.5)		
Passive smokers	4 (2.0)	4 (2.0)		
Alcohol consumption			17.627	<0.001^a^
Never drinkers	189 (94.5)	165 (82.5)		
Current drinkers	6 (3.0)	30 (15.0)		
Former drinkers	5 (2.5)	5 (2.5)		
Physical activity			2.077	0.354^a^
Never	56 (28.0)	63 (31.5)		
1-2 times/week	91 (45.5)	96 (48.0)		
≥3 times/week	53 (26.5)	41 (20.5)		
Sleep duration			2.547	0.111^a^
<6 h	45 (22.5)	59 (29.5)		
≥6 h	155 (77.5)	141 (70.5)		
Family history	6 (3.0)	2 (1.0)	1.148	0.284^b^
History of allergy	30 (15.0)	14 (7.0)	6.537	0.011^a^
Livestock husbandry	13 (6.5)	10 (5.0)	0.415	0.519^a^
Pet ownership	28 (14.0)	33 (16.5)	0.484	0.487^a^
Appendectomy	77 (38.5)	22 (11.0)	40.605	<0.001^a^
Breastfeeding			4.913	0.086^a^
Exclusive breastfeeding<3 months	37 (18.5)	28 (14.0)		
Exclusive breastfeeding≥3 months	108 (54.0)	97 (48.5)		
Mixed feeding	55 (27.5)	75 (37.5)		
Modes of delivery			0.219	0.640^a^
Vaginal delivery	150 (75.0)	154 (77.0)		
Cesarean delivery	50 (25.0)	46 (23.0)		
History of antibiotic use during childhood			0.342	0.952^a^
No	77 (38.5)	74 (37.0)		
Seldom:<3 times/year	55 (27.5)	54 (27.0)		
Frequent:≥3 times/year	20 (10.0)	19 (9.5)		
Unknown	48 (24.0)	53 (26.5)		
Childhood gastrointestinal infections			5.752	0.124^c^
No	112 (56.0)	108 (54.0)		
Seldom:<3 times/year	33 (16.5)	47 (23.5)		
Frequent: ≥3 times/year	7 (3.5)	2 (1.0)		
Unknown	48 (24.0)	43 (21.5)		
Measles, rubella and mumps during childhood			2.634	0.268^a^
No	117 (58.5)	125 (62.5)		
Yes	48 (24.0)	35 (17.5)		
Unknown	35 (17.5)	40 (20.0)		
NSAIDs, excluding aspirin			2.469	0.481^c^
Never	102 (51.0)	98 (49.0)		
Prior use (discontinued)	87 (43.5)	85 (42.5)		
Current use <5 years	8 (4.0)	15 (7.5)		
Current use ≥5 years	3 (1.5)	2 (1.0)		
Aspirin			6.485	0.090^c^
Never	141 (70.5)	158 (79.0)		
Prior use (discontinued)	51 (25.5)	31 (15.5)		
Current use <5 years	7 (3.5)	9 (4.5)		
Current use ≥5 years	1 (0.5)	2 (1.0)		
Oral contraceptives			0.613	0.736^c^
Never	57 (87.7)	94 (84.7)		
Prior use (discontinued)	7 (10.8)	16 (14.4)		
Current use <5 years	0	0		
Current use ≥5 years	1 (1.5)	1 (0.9)		
Parasitic infections	23 (11.5)	15 (7.5)	1.861	0.173^a^
Intestinal discomfort	176 (88.0)	25 (12.5)	228.016	<0.001^a^
Perianal complications	121 (60.5)	3 (1.5)	162.740	<0.001^a^

^a^ Pearson’s chi-square test.

^b^ Continuity correction.

^c^ Likelihood ratio chi-square.

^d^ Fisher’s exact test.

### 3.6 Diagnostic delay

#### 3.6.1 Determinants of diagnostic delay in Crohn’s disease

**FIGURE 4:**
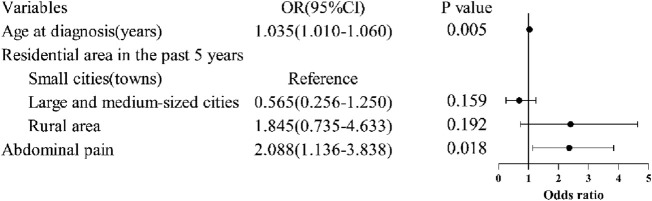
Multivariable analysis of factors influencing diagnostic delay in Crohn’s disease. Multivariable logistic regression confirmed advanced age at diagnosis as an independent risk factor, while abdominal pain exhibited protective effects against diagnostic delay.

In this cohort of 200 CD patients, diagnostic delay (median duration: 23 months, IQR 12–48) occurred in 48.0% (n = 96). Univariate screening identified advanced age at diagnosis, recent residential area (past 5 years), and absence of abdominal pain as significant predictors of delayed diagnosis (Tables 1, 2). Multivariable logistic regression confirmed advanced age at diagnosis as an independent risk factor (OR = 1.035 per additional year, 95% CI: 1.010–1.060, *P* = 0.005), while abdominal pain exhibited protective effects against diagnostic delay (OR = 2.088, 95% CI: 1.136–3.838, *P* = 0.018), suggesting symptom-driven diagnostic acceleration in symptomatic presentations (Figure 4).

#### 3.6.2 Temporal correlates of diagnostic delay in Crohn’s disease

**FIGURE 5:**
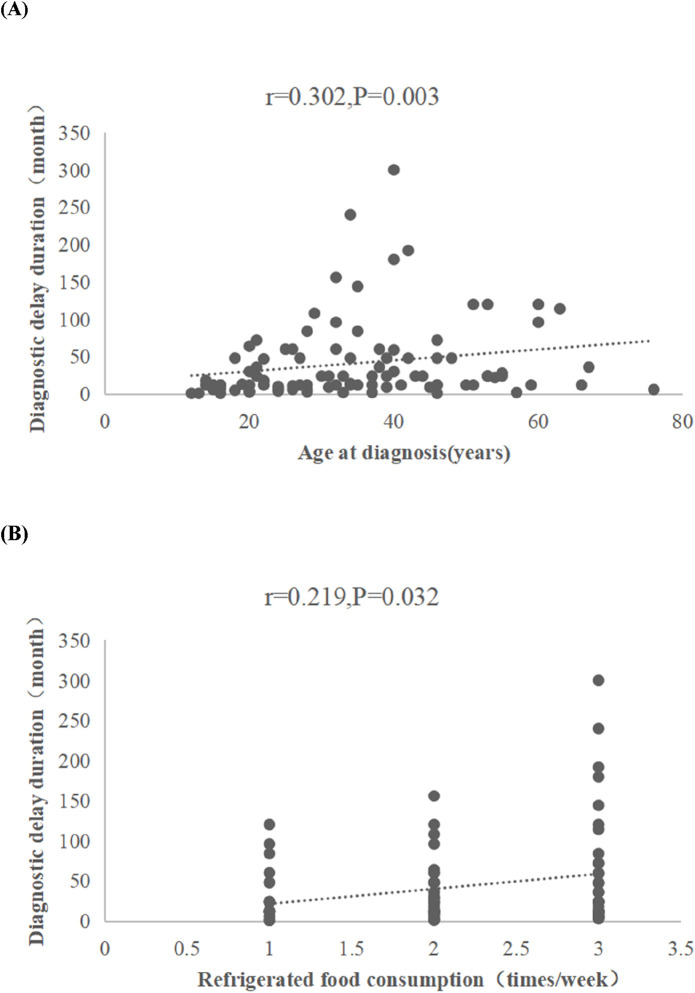
Spearman’s rank correlation analysis between diagnostic delay duration and relevant variables. Scatter plots show correlations related to diagnostic delay duration. **(A)** This scatter plot graphs age at diagnosis against diagnostic delay, demonstrating a significant positive correlation. **(B)** This scatter plot graphs refrigerated food consumption frequency against diagnostic delay, demonstrating a positive correlation.

Spearman’s rank correlation analysis revealed significant positive associations between diagnostic delay duration and both advanced age at diagnosis (r = 0.302, *P* = 0.003) and frequent consumption of refrigerated foods (r = 0.219, *P* = 0.032), while educational attainment and other dietary behaviors showed no statistically meaningful correlations (Figure 5).

#### 3.6.3 Univariate logistic regression analysis of intestinal obstruction, perforation, and surgical intervention

Univariate logistic regression analysis demonstrated no statistically significant associations between diagnostic delay duration and complications including intestinal obstruction (OR = 1.12, 95% CI: 0.89–1.41), perforation (OR = 0.97, 95% CI: 0.72–1.30), or surgical intervention rates (OR = 1.08, 95% CI: 0.93–1.25), with all comparisons exceeding the α = 0.05 threshold (Table 5).

**TABLE 5:** Univariate logistic regression analysis of intestinal obstruction, perforation, and surgical intervention.

Variables	OR	95%CI	*P* Value
Intestinal obstruction	1.182	0.829–1.684	0.356
Intestinal perforation	1.467	0.871–2.471	0.150
Surgical intervention	1.350	0.949–1.921	0.095

## 4 Discussion

Crohn’s disease (CD), a chronic idiopathic inflammatory bowel disorder, exhibits rising incidence in China, though epidemiological data remain scarce in coastal regions. This study reports a crude CD prevalence of 17.48/10^5^ and incidence of 2.30/10^5^ in Yancheng, a coastal city—substantially higher than rates in Hong Kong SAR (1.25/100,000) (Ng et al., 2013), Guangdong Province (1.09/100,000) (Zeng et al., 2013), Taiwan region (0.81/100,000) (Kuo et al., 2024), Wuhan City (0.51/100,000) (Jiang et al., 2007), Chengdu City (0.14/100,000),Xi’an City (0.07/100,000 (Ng et al., 2013), yet below Western benchmarks (Australia: 29.3/10^5^; Canada: 319.0/10^5^) (Ng et al., 2017). This epidemiological pattern suggests coastal regions may constitute disease hotspots influenced by distinct environmental determinants.

The cohort demonstrated male predominance (M:F = 2.07:1), and median diagnosis age of 29 years (IQR 20–39). This demographic profile aligns with global epidemiological patterns of CD in terms of gender distribution and age-stratified incidence. The observed male preponderance may be mediated through sex-specific environmental exposures and differential sex hormone profiles (Tshikudi et al., 2025). The study cohort demonstrated geographical clustering in Tinghu District (26.0% vs. 3.5% in Xiangshui County), likely reflecting imbalances between healthcare resources and economic development. Disease phenotypes aligned with global patterns: ileocolonic involvement (L3:47.0%), non-stricturing, non-penetrating disease behavior (B1:55.0%), and frequent perianal complications (60.5%). Epidemiological studies have demonstrated that CD patients with concomitant perianal complications exhibit 3- to 4-fold elevated incidence and prevalence rates compared to the general population (H Everhov et al., 2025). Biologic therapy predominance (89.0%) underscores treatment intensification trends, while simultaneously indicating potential enrollment bias toward patients with more severe disease phenotypes or treatment-refractory profiles.

The culinary profile of Yancheng region is characterized by a high-sodium and capsaicin-rich dietary regimen, with preserved foods—including traditional fermented sausages and estuarine mud snails (bullacta exarata)—occupying a prominent nutritional niche due to their organoleptic distinctiveness and superior preservation properties under humid subtropical conditions. Contrary to established dietary risks (Papadimitriou et al., 2025), multivariable analysis identified protective associations for milk (OR = 0.389), fried food (OR = 0.329), preserved foods (OR = 0.357), alcohol consumption (OR = 0.176) and spicy intake (OR = 0.241). These paradoxical findings may reflect reverse causation: post-diagnosis dietary modifications (e.g., self-imposed elimination diet (Tingting et al., 2022)), which may distort retrospective exposure assessments. Mechanistically plausible hypotheses exist, including the potential protective effects of probiotics in dairy products and mucosal adaptation to dietary spices. Notably, seafood showed null associations, potentially obscured by similar behavioral adjustments. Female sex, lower occupational physical demands, higher educational attainment, and elevated socioeconomic status conferred additional protection, potentially mediated by enhanced health literacy and proactive healthcare-seeking behaviors. Animal-derived proteins from fish and poultry, as key components of the Mediterranean diet, provide essential amino acids, vitamin B12, and bioavailable heme iron. Their consumption is inversely associated with Crohn’s disease (CD) incidence, potentially mediated through maintaining metabolic homeostasis and preserving muscle mass (Guasch-Ferré and Willett, 2021). The observed dietary paradoxes likely reflect reverse causation bias introduced by post-diagnosis dietary modifications, underscoring the necessity for large-scale prospective cohorts with longitudinal dietary monitoring to disentangle true etiological relationships from behavioral adaptations. Rural residency was identified as an independent risk factor for CD, with exposure pathways likely mediated through environmental contaminants in water supplies, barriers to healthcare access, and insufficient health awareness. Our cohort analysis demonstrates a significantly elevated risk of Crohn’s disease among atopic individuals (OR = 3.801, 95%CI:1.660-8.703), consistent with other study (Virta et al., 2013) Shared environmental-immunogenetic mechanisms underlie both allergic diseases and IBD. Mast cells serve as key effector cells in allergic responses, where allergen-specific IgE crosslinking triggers degranulation and release of histamine/leukotrienes. Critically, these pro-inflammatory mediators similarly contribute to Crohn’s disease pathogenesis (Kanchan et al., 2021). While an epidemiological link between appendectomy and subsequent CD diagnosis was observed, this finding is likely confounded by diagnostic misclassification. The considerable clinical mimicry of incipient CD and acute appendicitis often results in erroneous appendectomies prior to definitive IBD diagnosis. Early-onset intestinal symptoms and perianal complications may serve as clinical harbingers of elevated Crohn’s disease risk. It is reported that up to 30% of CD patients present with perianal complications at diagnosis, while up to 17% exhibit isolated perianal pathology preceding formal diagnosis (Eglinton et al., 2012). Consequently, vigilance for underlying Crohn’s disease is warranted in patients presenting with isolated perianal pathology, enabling timely diagnostic confirmation and reducing therapeutic delays.

Therapeutic efficacy during CD’s early “window of opportunity” is critically compromised by diagnostic delays. In anti-tumor necrosis factor (TNF) therapy efficacy trials, a substantial proportion of patients exhibit primary nonresponse, potentially attributable to prolonged diagnostic delays and subsequent irreversible intestinal architectural damage (Spencer et al., 2002). However, diagnostic delay during Crohn’s disease management has garnered increasing research attention, primarily categorized into patient-dependent delay and healthcare system-related delay. Diagnostic delays plagued 48% of cases (median 23 months) in this study, substantially exceeding the median durations reported in other countries (e.g., France: 5 months (Nahon et al., 2016), Switzerland: 9 months (Schoepfer et al., 2013), United States: 9.5 months (Furfaro et al., 2014)). Multivariable analysis identified advanced age at diagnosis (OR = 1.035) as an independent risk factor for diagnostic delay due to atypical presentations mimicking ischemic/medication-induced colitis (Jia-ming and Hong, 2018). Notably, patients with late-onset disease exhibit a significantly elevated risk of adverse clinical outcomes (Singh et al., 2024). Yancheng’s diagnostic delay prevalence reflects systemic healthcare disparities and inadequate diagnostic infrastructure (limited fecal calprotectin/microbiome testing). The current distribution of IBD specialists in China remains suboptimal, with clinicians predominantly concentrated in developed regions, resulting in a critical shortage of specialized personnel in primary care hospitals. According to data from the China Crohn’s and Colitis Foundation (CCCF), the Yancheng region has only six IBD-certified gastroenterologists, with four at Yancheng NO.1 People’s Hospital and two at Yancheng TCM Hospital. These systemic deficiencies mirror broader challenges in China’s IBD care cascade. Urgent reforms—specialist training programs, tiered referral networks, and point-of-care biomarker deployment—are imperative to mitigate complication risks in late-onset CD populations. Contrastingly, abdominal pain (OR = 2.088) emerged as a protective determinant, potentially mediated through severe pain-induced prompt medical consultation that expedited diagnostic confirmation.

Spearman’s analysis demonstrated significant positive associations between diagnostic delay duration and both age at diagnosis (r = 0.302, *P* = 0.003) and frequency of refrigerated food consumption (r = 0.219, *P* = 0.032), suggesting that advanced age and frequent refrigerated dietary exposures may elevate risks of delayed diagnosis. The latter association may be linked to intestinal dysbiosis induced by prolonged consumption of refrigerated food (e.g., leftover dishes, cold beverages) or exacerbation of mucosal damage from thermal stress due to low-temperature exposure, necessitating validation of these mechanistic pathways through prospective studies. Under the cold-chain hypothesis, advancements in industrial and domestic refrigeration have led to frequent human exposure to psychrotrophic bacteria capable of proliferating in chilled environments. Notably, *Yersinia* species may exacerbate enteric inflammation in genetically susceptible individuals. However, given their typically low pathogenicity, the reported infection rates remain disproportionately low relative to exposure prevalence, potentially resulting in diagnostic delays (Hugot et al., 2021). Chinese cohorts reveal protective effects against CD in households without refrigerators (Shi et al., 2008), reinforcing temperature-dependent microbial dysbiosis as a plausible mechanism. Furthermore, diagnostic delay duration demonstrates significant associations with exacerbated intestinal architectural damage, increased necessity for CD-related surgical interventions, and elevated risks of adverse long-term prognostic outcomes (Schoepfer et al., 2013; Jia et al., 2018). Not with standing the absence of statistically significant associations between diagnostic delay and risks of intestinal obstruction, intestinal perforation, or surgical intervention in univariate analyses within this cohort, these null findings may be attributable to limitations in statistical power or residual confounding. Subsequent investigations with expanded sample sizes are warranted to clarify these relationships.

The substantial epidemiological burden of Crohn’s disease in coastal Yancheng—marked by elevated incidence rates and protracted diagnostic delays—underscores the urgency of region-specific prevention strategies. While our findings illuminate novel environmental-dietary interactions and healthcare system vulnerabilities, validation through population-based cohorts remains imperative to address coastal China’s epidemiological data gap. Priority interventions must establish multidisciplinary task forces integrating fecal biomarker screening, specialist telemedicine networks, and community-based cold-chain food safety initiatives, particularly targeting aging populations and high-risk dietary patterns. Concurrently, national IBD registries should be expanded to monitor delayed diagnosis consequences on long-term disability-adjusted life years (DALYs), ensuring equitable resource allocation across rapidly developing regions.

## 5 Limitations

This hospital-based retrospective design inherently carries selection bias, limiting generalizability to population-level CD epidemiology in coastal China. The findings primarily reflect CD incidence patterns within the Yancheng region. Recall bias in dietary/lifestyle assessments—particularly regarding pre-diagnosis exposures—and unmeasured environmental confounders (industrial pollutants, waterborne contaminants) may distort observed associations. Besides, changes in relevant indicators during disease progression may have some impact on observed outcomes such as treatment modalities. Furthermore, the lack of granular treatment data (biologic dosing schedules, concomitant immunomodulators) and longitudinal outcome measures precludes analysis of therapeutic durability and complication trajectories. This study primarily provides a descriptive analysis of region-specific epidemiological characteristics of CD, without delving into underlying pathological mechanisms; its preliminary nature warrants further in-depth investigation. These limitations necessitate future validation through prospective population-based cohorts integrating multi-omics profiling (metagenomics, metabolomics) with geospatial environmental monitoring to elucidate coastal CD pathogenesis.

## 6 Conclusion

The Yancheng region exhibits higher CD prevalence and incidence rates compared to national averages, with pronounced diagnostic delays. These findings highlight the need for targeted interventions to improve early diagnosis and mitigate healthcare burdens.

## Data Availability

The raw data supporting the conclusions of this article will be made available by the authors, without undue reservation.
